# Primary Motor Cortex Representation of Handgrip Muscles in Patients with Leprosy

**DOI:** 10.1371/journal.pntd.0003944

**Published:** 2015-07-23

**Authors:** Vagner Wilian Batista e Sá, Maria Katia Gomes, Maria Luíza Sales Rangel, Tiago Arruda Sanchez, Filipe Azaline Moreira, Sebastian Hoefle, Inaiacy Bittencourt Souto, Antônio José Ledo Alves da Cunha, Ana Paula Fontana, Claudia Domingues Vargas

**Affiliations:** 1 Núcleo de Pesquisas em Fisioterapia, Universidade Castelo Branco, Rio de Janeiro, Rio de Janeiro, Brazil; 2 Programa de Pós-Graduação em Clínica Médica, Hospital Universitário Clementino Fraga Filho e Departamento de Medicina de Família e Comunidade/Faculdade de Medicina da Universidade Federal do Rio de Janeiro, Rio de Janeiro, Rio de Janeiro, Brazil; 3 Laboratório de Neurobiologia II, Instituto de Biofísica Carlos Chagas Filho, Universidade Federal do Rio de Janeiro, Rio de Janeiro, Rio de Janeiro, Brazil; 4 Cognitive and Behavioral Neuroscience Unit and Neuroinformatics Workgroup, D'Or Institute for Research and Education, Rio de Janeiro, Brazil; 5 Instituto de Neurologia Deolindo Couto da Universidade Federal do Rio de Janeiro, Rio de Janeiro, Rio de Janeiro, Brazil; Emory University, UNITED STATES

## Abstract

**Background:**

Leprosy is an endemic infectious disease caused by *Mycobacterium leprae* that predominantly attacks the skin and peripheral nerves, leading to progressive impairment of motor, sensory and autonomic function. Little is known about how this peripheral neuropathy affects corticospinal excitability of handgrip muscles. Our purpose was to explore the motor cortex organization after progressive peripheral nerve injury and upper-limb dysfunction induced by leprosy using noninvasive transcranial magnetic stimulation (TMS).

**Methods:**

In a cross-sectional study design, we mapped bilaterally in the primary motor cortex (M1) the representations of the hand flexor digitorum superficialis (FDS), as well as of the intrinsic hand muscles abductor pollicis brevis (APB), first dorsal interosseous (FDI) and abductor digiti minimi (ADM). All participants underwent clinical assessment, handgrip dynamometry and motor and sensory nerve conduction exams 30 days before mapping. Wilcoxon signed rank and Mann-Whitney tests were performed with an alpha-value of p<0.05.

**Findings:**

Dynamometry performance of the patients’ most affected hand (MAH), was worse than that of the less affected hand (LAH) and of healthy controls participants (p = 0.031), confirming handgrip impairment. Motor threshold (MT) of the FDS muscle was higher in both hemispheres in patients as compared to controls, and lower in the hemisphere contralateral to the MAH when compared to that of the LAH. Moreover, motor evoked potential (MEP) amplitudes collected in the FDS of the MAH were higher in comparison to those of controls. Strikingly, MEPs in the intrinsic hand muscle FDI had lower amplitudes in the hemisphere contralateral to MAH as compared to those of the LAH and the control group. Taken together, these results are suggestive of a more robust representation of an extrinsic hand flexor and impaired intrinsic hand muscle function in the hemisphere contralateral to the MAH due to leprosy.

**Conclusion:**

Decreased sensory-motor function induced by leprosy affects handgrip muscle representation in M1.

## Author Summary

Leprosy is an endemic disease caused by *Mycobacterium leprae* that predominantly attacks both skin and peripheral nerves, resulting in persistent distal hand atrophy and the loss of sensory and autonomic functions. In this study, we employ a noninvasive tool named transcranial magnetic stimulation (TMS) to map the handgrip muscle representation in the primary motor cortex of patients affected by leprosy. The findings of this study support that the decrease or loss of sensory afferent neurons and/or impairment in the strength of peripheral muscles in the ulnar/median territory verified in leprosy patients alters the handgrip function leading to cortical motor reorganization in the corresponding affected hand muscles.

## Introduction

Leprosy, also known as Hansen's disease, is a chronic human granulomatous bacilliferous infection caused by the obligate intracellular organism *Mycobacterium leprae* [1]. Leprosy continues to be an important health problem worldwide, particularly in India, Brazil, Democratic Republic of Congo, Tanzania, Nepal, Mozambique, China and Nigeria [2,3]. The bacillus has a predisposition to infect cutaneous and peripheral nervous tissues, which allows infiltration into Schwann cells, resulting in nerve inflammation, most frequently in the eyes, hands and feet. This causes partial or total loss of sensory, motor and autonomic functions in the territory of the affected nerve resulting in skin anesthesia and dryness, as well as a decrease in proprioception and muscle paresis/hypotrophy [4].

Nerve damage may happen before, during or after treatment with the multidrug therapy (MDT) recommended by World Health Organization. In other words, even after bacteriological cure, leprosy can cause permanent physical disabilities and deformities. The installation of those deformities contributes to social exclusion, psychological disorders, and self-stigma, as has been recorded in studies about social participation and quality of life. Since 1985, 14 million individuals have received MDT [5]. Despite these efforts, every year, many patients develop upper limb disabilities and are in need of rehabilitation services to control the chronic consequences of neural damage, such as claw hand, neuropathic pain and burns, requiring technical and scientific advances and a deeper understanding of the outcomes of either short or long-term rehabilitation.

A considerable amount of literature has been published on leprosy. Several studies have focused on understanding the basic aspects of leprosy, such as genetics [6, 7], physiology of the bacilli [8,9], biological markers [10], kinesiology and biomechanical factors [11,12,13], and public health concerns [14,15]. Until recently, however, there has been little discussion about the relationship between the peripheral upper-limb dysfunction caused by leprosy and changes in the motor cortex [16,17]. This phenomenon, known as plasticity, refers to the physiological and structural changes that occur in the central nervous system over time.

The adult brain is capable of profound plasticity after peripheral lesions [18,19,20,21,22,23,24]. The potential for peripheral nerve injury to reorganize motor cortical representations was initially investigated in animal models [25]. Motor nerve injury is sufficient to produce changes in the primary motor cortex (M1) of mammals, these changes appearing as early as a few hours, days and even months after peripheral nerve injury [23,26].

Motor cortex plasticity has also been demonstrated after peripheral lesions caused by amputation and/or phantom pain [27,28,29,30], upper-limb muscle reconstruction and nerve transfer [21,31,32], uni and bilateral heterotopic hand transplantation [33,34,35], peripheral immobilization [36] and focal dystonia [19]. In most cases stated above, plasticity was accessed by means of transcranial magnetic stimulation (TMS) mapping.

It remains unknown if and how the human motor cortex reorganizes after the typical gradual peripheral nerve damage provoked by leprosy. In the present study, we used single-pulse TMS mapping to evaluate motor organization in M1 after MDT in adult chronic leprosy patients with persistent hand disabilities. We hypothesized that patients with chronic damage in upper limb peripheral nerve caused by leprosy could present changes in corticospinal excitability as well as in hand grip muscle representation organization in M1.

## Methods

### Design

This study employed a cross-section design where a transcranial magnetic stimulation (TMS) mapping protocol was used to evaluate the cortical representation of selected hand muscles in M1 contralateral and ipsilateral to the most affected hand in chronic leprosy patients and healthy subjects in Hospital Federal Clementino Fraga Filho, RJ, Brazil, over the period of 2009 to 2013.

### Ethics Statement

All participants provided written, informed consent, consistent with the Declaration of Helsinki. The Human Research Ethics Committee (CEP-HUCFF/UFRJ from the Universidade Federal do Rio de Janeiro, UFRJ, under registry 143/09) approved this study.

### Participants

Six right-handed adult patients with chronic leprosy, grade 2 disability (4 males; 31.2 ± 4.7 years; mean age ± SD; Table 1) and 6 healthy controls, matched in gender and handedness (4 males; 27.2 ± 4.6 years), participated in this study. Handedness was determined using the revised Edinburgh Handedness Inventory [37]. Inclusion criteria were: both gender with long-term leprosy waiting for surgery to correct claw deformities in hands, with age between 18 to 45 years old. The exclusion criteria where: previous fracture in the upper extremity, use of central nervous system medications, demyelinating disorders, history of neurological deficits, stroke, diabetes, systemic disease or migraine headache, cardiac pacemaker placement, osteoarthritis, history of specific repetitive motor activity or any putative adverse reaction to TMS [38,39,40].

**Table 1:** Clinical features of patients with leprosy's disease.

Patients N = 6	Gender	Age (years)	Leprosy Classification	Disease Duration (years)	Most Affected Hand	Affected Nerves (ENMG)	Handedness
**P1**	M	31	MB	6	Right	Ulnar median	Right
**P2**	F	30	MB	7	Right	Ulnar median	Right
**P3**	F	39	PB	20	Left	Ulnar	Right
**P4**	M	32	MB	5	Left	Ulnar median	Right
**P5**	M	31	MB	4	Left	Ulnar median	Right
**P6**	M	24	MB	3	Left	Ulnar median	Right

Note: ENMG: electroneuromyography; F: female; M: male; MB: multibacillary leprosy; PB: paucibacillary leprosy.

### Clinical Assessment

Two independent evaluators conducted a clinical assessment protocol for all participants, including anamnesis interview and active search for other information in the medical records.

### Dynamometry (Grip Strength-Handgrip)

Dynamometry testing required all measurements to be taken with subjects sitting comfortably on a chair with their hand resting on an armrest and feet flat on the floor. The volunteers held a digital dynamometer (EMG System do Brasil) with the shoulder and wrist at a neutral position with the forearm supported and the elbow positioned at 90 degrees [41,42,43]. The importance of maintaining this position was explained to the subjects and repeated in both hands. In some cases, the evaluator assisted the patients to maintain the device in the proper position. The trials began with 60 s. of rest, after which the participants were asked to maintain maximal grip contraction for six seconds. A mean of three trials was collected with intervals of 60 s. between trials. The first and last second of each trial were discarded. To ensure maximum effort, a verbal cue was given to the subject while he performed the test.

### Electroneuromyography Procedure (ENMG)

Leprosy neuropathy, despite being primarily demyelinating, frequently leads to axonal loss. Nerve conduction studies are considered the most objective method of assessing nerve function [44] to confirm the diagnosis/prognostic of neuropathy in leprosy patients. Neurophysiological examination of the nerves frequently shows that once axonal loss has been installed, nerve function is little affected by inflammatory, immune and/or bacterial events since chronic neuropathy has been established, inevitably leading to the well-known leprosy sequelae occurring at any time before and/or after leprosy diagnosis [45].

Patients in this study were examined in a nerve conduction study approximately 30 days before the TMS mapping. The ENMG protocol for the study of motor conduction of median and ulnar nerves was performed. The amplitude of the compound muscle action potentials (CMAP) as well as the latencies and motor conduction velocity (MCV) were measured. For the sensory conduction study in the same nerves, the compound sensory action potential amplitude (CSAP), distal latency and velocity of sensory conduction velocity (SCV) were measured. The skin temperature was measured and maintained above 32 degrees Celsius.

### Preparation for TMS Mapping

Prior to the TMS session, an image of the head of every subject was obtained using magnetic resonance imaging (MRI) using a neuronavigation system (3space Fastrack–Polhemus Isotrack II) to ensure the accurate positioning of the TMS coil. At the beginning of the experiment, the 3D location of 200 points on the scalp was measured using an electromagnetic position sensor to co-register the MRI with the actual position of the subject’s head [46]. A plastic cap with grid marks spaced at 1 cm intervals was secured in position to serve as a reference for reproducible coil placement and external orientation.

During the experiment, subjects remained awake, seated in a plastic comfortable chair with pillows placed under the forearms/hands, and his/her jewelry, glasses, watches and other potentially conducting or magnetic objects worn on the head or arms were removed to prevent interactions with the magnetic field, consistent with a TMS guide [39]. The skin surface over the forearm/hand muscles was washed, shaved and abraded with alcohol at 70% until an erythemic response appeared. To ensure consistent surface electromyography (sEMG) electrodes placement, the participant’s forearm/hand was measured with meter tape in each testing session. When possible, during all data collection, a manual muscle test was employed to isolate the target muscles, including the flexor digitorum superficialis (FDS), abductor pollicis brevis (APB), first dorsal interosseous (FDI) and abductor digiti minimi (ADM), was conducted to determine the optimal placement of the electrodes [47,48].

Surface EMG recordings were obtained using surface 8 mm, Ag/AgCl electrodes (Medtronic Adhesive Disposable Surface Electrodes) placed on the skin over the muscle bellies, and the centers of the electrodes were placed approximately 1.5 cm apart. A ground electrode was placed ipsilaterally above the epicondylus, laterally at the elbow joint. Surface EMG signals were amplified and band pass filtered (1–5000 Hz, Biopac MP150 Systems Inc.). The signal was subsequently digitized at a sampling rate of 15.000 Hz (A/D converter National Instruments—LABVIEW 7.0) and stored on a desktop computer for offline analysis. Custom-made MATLAB software (10—Mathworks, Inc., Massachusetts, USA) was used to measure the latency and peak-to-peak MEP amplitudes. The MEPs mean amplitudes recorded at each stimulated point was subsequently calculated and projected onto the brain to create a cortical muscle representation map. We visually inspected the EMG profiles to ensure that all muscles were electrically silent for each TMS pulse. When it was not the case, the trial was rejected and stimulation at that point was repeated.

### TMS Procedure

TMS mapping of four hand target muscles was performed using a MagVenture-MagPro R30 (Tonica Elektronik A/S, Denmark) connected to a figure-of-eight cooled coil (wing diameter = 75 mm; peak magnetic field strength 2.2 T; peak electric field strength 660 V/m, biphasic pulse). The elicited electric field was directed in the lateral to medial direction with the coil held in a tangential position with the handle perpendicular to the midsagittal line.

Before mapping, the FDS hot spot (position on the scalp where FDS muscle responses could be reliably evoked with the lowest stimulator intensity and highest peak-to-peak amplitudes) was located. Subsequently the resting motor threshold, which corresponds to the minimal intensity of stimulation at the hotspot eliciting MEPs larger than 50 μV in at least 50% of 10 trials, was determined according to the threshold-hunting paradigm [49]. Once these parameters were determined, the simultaneous mapping of four target muscles was performed, with the output intensity adjusted to 120% of the resting motor threshold. A total of 10 consecutive pulses were delivered at each stimulated site of the grid, beginning at the hotspot and moving in a spiral direction, with 3- to 5-second inter-pulse intervals. Mapping was complete when the locations adjacent to the active sites were identified as non-active/no MEP [21,38]. MEPs associated to muscle contractions were discarded.

After data collection, the average latency and amplitude of the MEPs for each site was calculated. The mean amplitude obtained per coordinate was then normalized per participant by dividing the mean amplitudes by the maximum mean amplitudes collected in the hotspots. The center of gravity (COG), map area (number of active sites) and muscle overlaps were measured from MEP normalized values. The COG was defined as the map location representing the amplitude-weighted center of the area of excitability [50]. The COG (*x*,*y*) coordinate was calculated as:

COG=[∑aixi/∑ai,∑aiyi/∑ai]

Where, *a*_*i*_ represents the mean amplitude, and *x*_*i*_ and *y*_*i*_ represents the stimulated coordinate position.

The Euclidean equation was applied to determine the distance between COG locations in the same hemisphere, whereas the map area was defined as sum of active sites with >50 μV/MEP. The muscle overlap parameter was defined as the TMS stimulation points that generated simultaneous MEPs in all four-target muscles [51,50].

### Data Analysis

Descriptive and nonparametric analyses were performed using STATISTICA 7.0 (StatSoft Inc.,Tulsa, USA) and GraphPad Prism 6 (GraphPad Software, Inc.,San Diego, USA). To study the handgrip (dynamometry), TMS map area and COG we used a Wilcoxon matched-pairs signed rank test. The Mann-Whitney test was chosen to analyze the TMS motor threshold and MEP amplitudes. For all statistical tests, the alpha level was set to p<0.05.

## Results

### Electroneuromyographic Data (ENMG)

The ENMG exam was used to assess the bilateral ulnar/median nerve conduction in leprosy patients. Table 2 presents the results of the evaluation of the patients’ sensory and motor potentials. All tested patients presented a severe\complete impairment (latency, amplitude and conduction velocity) of both motor and sensitive fibers of the ulnar nerve (innervating FDI and ADM muscles) in one of the limbs, which was thus defined as “the most affected hand (MAH)”. Furthermore, patients P1, P2, P4 and P6 showed a severe loss in the sensory component of the median nerve. Patient P1 also presented evidence of damage in the motor fibers of the median nerve. All the other patients presented normal values within the standardized ENMG thresholds for the median nerve. Indeed, the axonal injury caused by *M*.*leprae* courses with a distal to proximal pattern, with an initial ulnar nerve sensory impairment followed by injury in motor fibers as well, described in the literature as impairment of conduction of nerve impulse [52] and decreased amplitude of sensory-motor potentials [53]. If not diagnosed and treated in time the disease progresses towards affecting other nerves, such as the median.

**Table 2:** Electroneuromyography in leprosy (ENMG).

Patients /		MOTOR EVOKED POTENTIALS						SENSORY EVOKED POTENTIALS					
nerves		LATENCY (ms)		CMAP (μV)		MCV (m/s)		LATENCY (ms)		CSAP (μV)		SCV (m/s)	
		MAH	LAH	MAH	LAH	MAH	LAH	MAH	LAH	MAH	LAH	MAH	LAH
**P1**	**Ulnar**	A	P (7,2)	A	D (1,2)	A	D (41)	A	A	A	A	A	A
	**Median**	A	P (4,4)	A	N (7,5)	A	D (38)	A	A	A	A	A	A
**P2**	**Ulnar**	A	N (2,3)	A	N (4,8)	A	N (56)	A	A	A	A	A	A
	**Median**	N (3,6)	N (3,2)	N (6,4)	N (8,6)	N (55)	N (53)	A	A	A	A	A	A
**P3**	**Ulnar**	A	N (2,4)	A	N (7,9)	A	N (63)	A	N (2,0)	A	N (14)	A	N (50)
	**Median**	N (3,6)	N (3,8)	N (11,5)	N (6,8)	N (49)	N (58)	N (3,0)	N (2,9)	N (16)	N (16)	N (43)	N (45)
**P4**	**Ulnar**	P (5,8)	N (2,8)	D (0,6)	N (8,3)	##	N (51)	A	A	A	A	A	A
	**Median**	N (3,0)	N (3,2)	N (11,5)	N (10,6)	N (51)	N (50)	A	A	A	A	A	A
**P5**	**Ulnar**	A	N (3,0)	A	N (4,6)	A	N (59)	A	A	A	A	A	A
	**Median**	N (2,8)	N (2,8)	N (13,3)	N (6,7)	N (55)	N (49)	N (2,4)	A	N (11)	A	N (54)	A
**P6**	**Ulnar**	A	P (4,5)	A	P (1,3)	A	##	A	A	A	A	A	A
	**Median**	N (3,3)	N (3,5)	N (5,5)	N (5,3)	N (52)	N (48)	A	A	A	A	A	A

Note: CMAP: compound motor action potential amplitude; MCV: motor conduction velocity; CSAP: compound sensory action potential; SCV: sensory conduction velocity; MAH: most affected hand; LAH: less affected hand; A: absent; N: normal; P: prolonged; D: diminished; **: unevaluated

##: absent.

Reference values: MOTOR LATENCY (**ms**): median (APB), ≤4.2; ulnar (ADM) ≤3.4. CMAP (**μV**): median, ≥3.5; ulnar, ≥2.7.MCV (**m/s**): median, ≥48; SENSORY LATENCY(**ms)**: median\ulnar, ≤3.0; CSAP (**μV)**: median (first finger, ≥10; third finger, ≥15); ulnar (fifth finger, ≥8). SCV (**m/s**): median (first finger, ≥40; third finger, ≥45); ulnar (fifth finger, ≥40).

### Dynamometry (Grip Strength) Data

**Fig 1:**
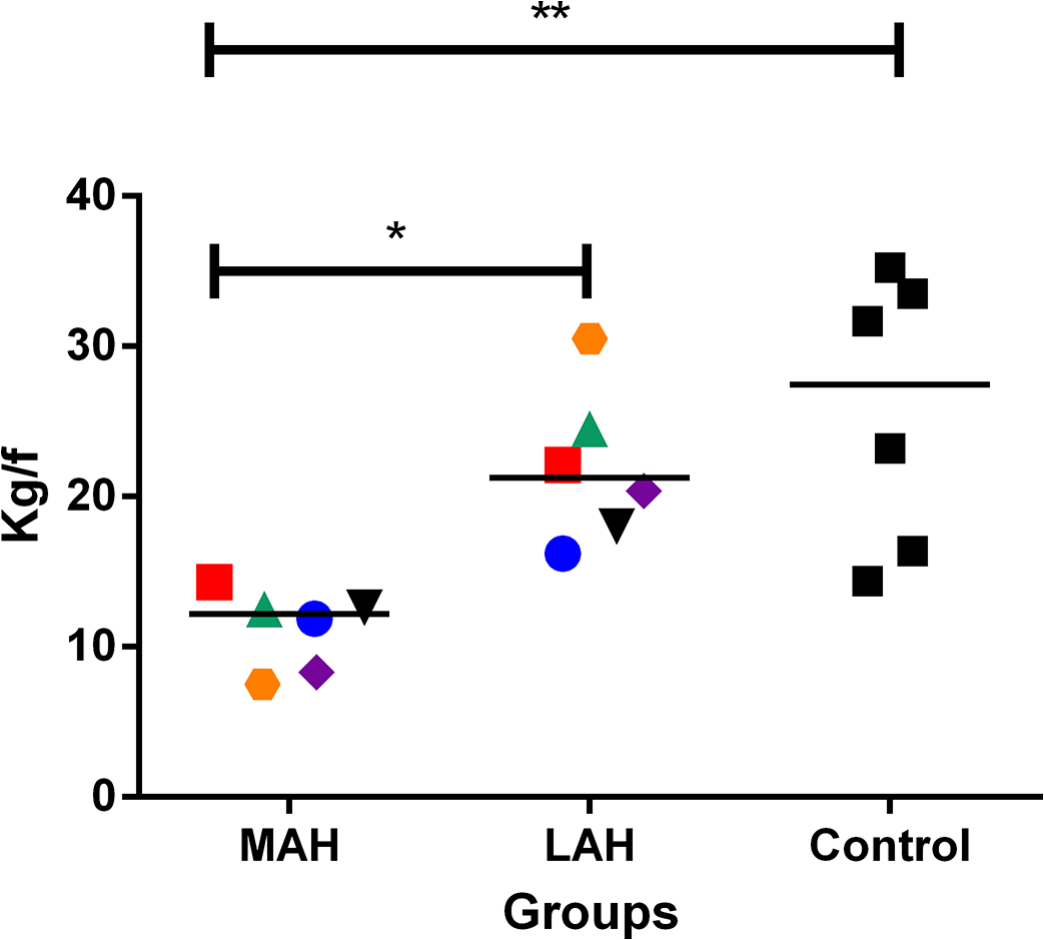
Handgrip assessment in leprosy group. Handgrip assessment. MAH: most affected hand; LAH: less affected hand; Control: average of grip strength in both upper-limbs (N = 6). P1(red square); P2(blue circle), P3(green triangle); P4 (purple rhombus); P5 (orange diamond); P6 (black triangle). The Wilcoxon matched-pairs signed rank test showed differences between MAH / LAH; p = 0.031*, and MAH / control group; p = 0.031**).

Firstly, we compared grip force in both upper-limbs of control subjects and found no significant difference (Wilcoxon test, p = 0.156). The average of the grip strength in both upper limbs of the control group (standard value) was then compared with that of patients. Lower grip strength was found for the MAH as compared to the control group (p = 0.031). Lower grip strength was also found in the MAH (p = 0.031) as compared with the less affected hand (LAH) in the patient’s group. These results are shown in Fig 1.

### Corticospinal Excitability

The percentage of TMS machine output (motor threshold), and the distance in time observed between the TMS stimulation artifact and the MEP onset (latency) were measured at the FDS muscle hotspot. Moreover, MEP average peak-to-peak (amplitude) was measured from the FDS, APB, FDI and ADM muscle hotspots. Table 3 shows individual values of these parameters obtained from patients and controls.

**Table 3:** TMS delivered on both hemispheres over the FDS hotspot. (Symbols retired.)

Patients					Controls		
N(6)	MT (%)	Amplitude (mV) 120% of MT	Latency (ms)	N(6)	MT (%)	Amplitude (mV) 120% of MT	Latency (ms)
	**CH / IH**	**CH / IH**	**CH / IH**		**MCH / MIH**	**MCH / MIH**	**MCH / MIH**
**P1**	62 / **	1,16 / **	22,0 / **	**C1**	58 / 40	0,31 / 0,36	19,5 / 18,6
**P2**	61 / **	1,31 / **	17,3 / **	**C2**	56 / 58	0,41 / 0,62	16,4/ 17,3
**P3**	68 / 80	1,04 / 0,26	17,8 / 19,6	**C3**	50 / 55	0,65 / 0,46	16,5 / 18,9
**P4**	72 / 79	0,37 / 0,57	19,5 / 21,7	**C4**	63 / 60	0,28 / 0,31	20,6 / 20,4
**P5**	50 / 60	0,79 / 1,21	21,8 / 19,7	**C5**	61 / 50	0,47 / 0,38	19,6 / 18,5
**P6**	66 / 72	0, 52 / 0,25	22,7 / 21,4	**C6**	45 / 45	0,25 / 0,44	23,5 / 20,0
**Median**	64,0 / 75,5	0,92 / 0,42	20,7 / 20,6		57,0 / 52,5	0,36 / 0,41	19,5 / 18,8

Note: Individual values of motor threshold, latency and MEP amplitude values in patients (P1 to P6) and paired control subjects (C1 to C6). Amplitude (in **milivolts**) at 120% of the motor threshold; MEP: motor evoked potential; MT: motor threshold; CH: hemisphere contralateral to the most affected hand; IH: hemisphere ipsilateral to the most affected hand; FDS: flexor digitorum superficialis muscle; latency (in **milliseconds**); MCH: matched contralateral hemisphere; MIH: matched ipsilateral hemisphere

** not tested.

**Fig 2:**
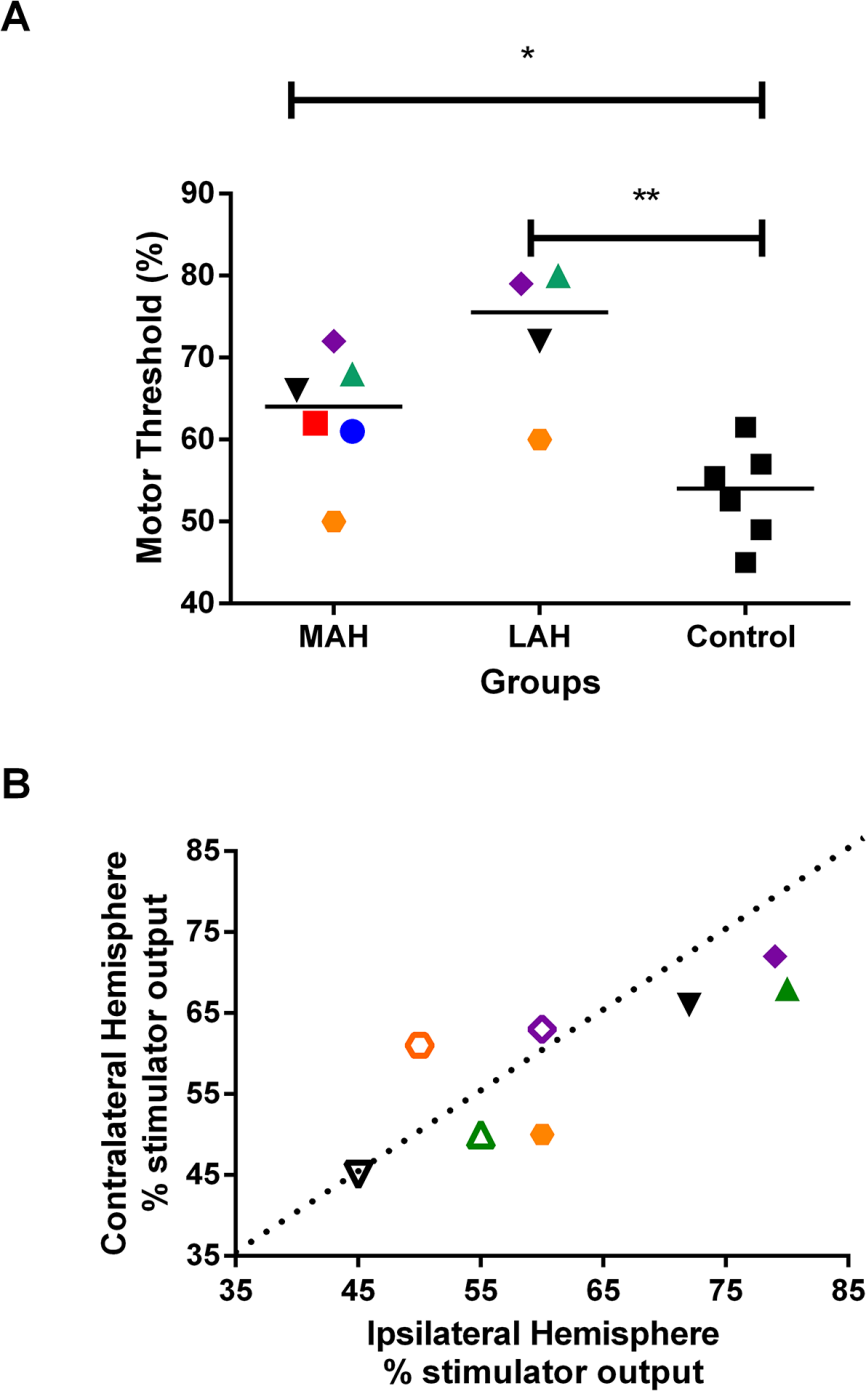
Resting motor threshold for flexor digitorum superficialis (FDS). **2A.** MAH: most affected hand; LAH: less affected hand; Control: average of bilateral motor threshold (N = 6). Patients´ symbols P1 (red square); P2 (blue circle), P3 (green triangle); P4 (purple rhombus); P5 (orange diamond); P6 (black triangle). For the P3 to P6 patients, the resting motor threshold for the FDS muscle were higher on less affected hand than the most affected hand. The Mann-Whitney test showed differences between MAH / control; p = 0.041*, and LAH / control; p = 0.019**. Similar symbols represent matched individuals. **2B.** Resting motor threshold for FDS on the contralateral hemisphere to the most affected hand and ipsilateral hemisphere in P3 to P6 patients (closed symbols) and matched subjects (open symbols). A patient (P5) demonstrates a different pattern showing higher motor threshold in ipsilateral hemisphere than their control subject.

We found a significant difference in motor threshold (MT) for the FDS muscle between the MAH and the control group (p = 0.041) as well as between the LAH and the control group (p = 0.019, Fig 2A). When the MT of the hemisphere contralateral to the MAH was plotted as function of the ipsilateral hemisphere per subject, it was clear that the MT was lower in the contralateral than in the ipsilateral hemisphere for the patients, whereas this parameter was fairly balanced in the control group. Accordingly, such interhemispheric bias is absent in healthy volunteers [54].

**Fig 3:**
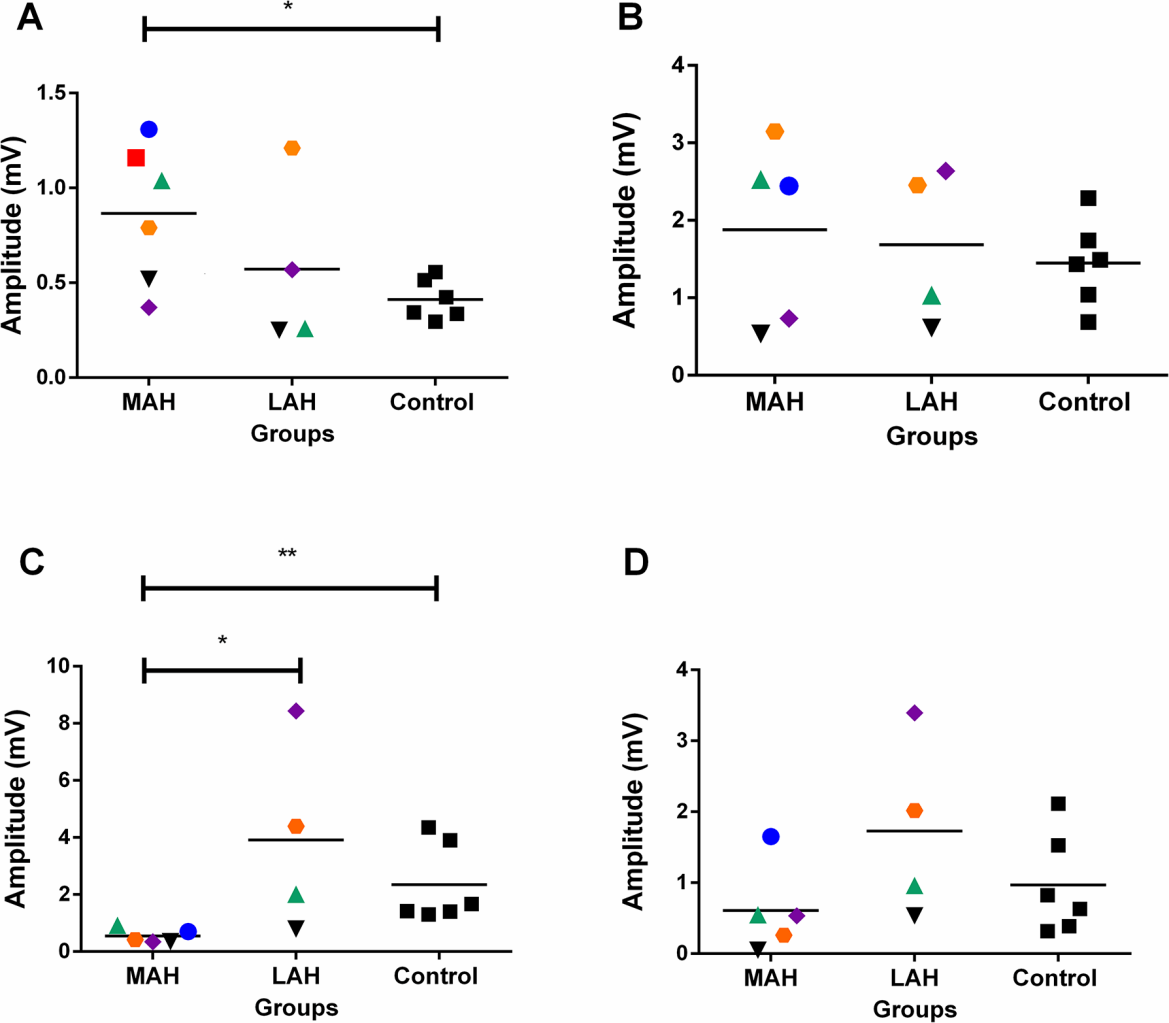
MEP amplitudes of the flexor digitorum superficialis (FDS), abductor pollicis brevis (APB), first dorsal interosseous (FDI), and abductor digiti minimi (ADM) muscles. MAH: Most affected hand. LAH: less affected hand. Control: Represents an average of amplitudes of both hemispheres in the control group. The Mann-Whitney test showed significant differences for (A) FDS (MAH x Control; *p = 0*.026*) and (C) FDI (MAH x LAH, p = 0.031*; MAH x control, p = 0.004**) muscles. The letters A, B, C and D represent the registered muscles Flexor Digitorum Superficialis (FDS), Abductor Pollicis Brevis (APB), First Dorsal Interosseous (FDI), and Abductor Digiti minimi (ADM), respectively. The average MEP amplitudes were higher in patients’ hemispheres than in healthy controls for FDS and APB muscles (Charts A and B, respectively).

Mann-Whitney test also showed significant differences for FDS amplitudes between the hemisphere contralateral to the MAH and the control group (p = 0.026), as well as for FDI amplitudes between the hemisphere contralateral to the MAH and those of the LAH (p = 0.031) as well as between those of the hemisphere contralateral to the MAH and the control group (p = 0.004, Fig 3).

### Map Area Overlaps

For each tested muscle, a map area was defined as the number of active sites with MEP average amplitudes equal or higher than 50 μV. In Patient (P1), diagnosed with serious right upper limb nerve damage, no MEP response could be elicited in the tested intrinsic muscles (APB, FDI and ADM), even with 99% of the TMS machine output. Great variability in map area was observed for all other patients and control subjects.

**Fig 4:**
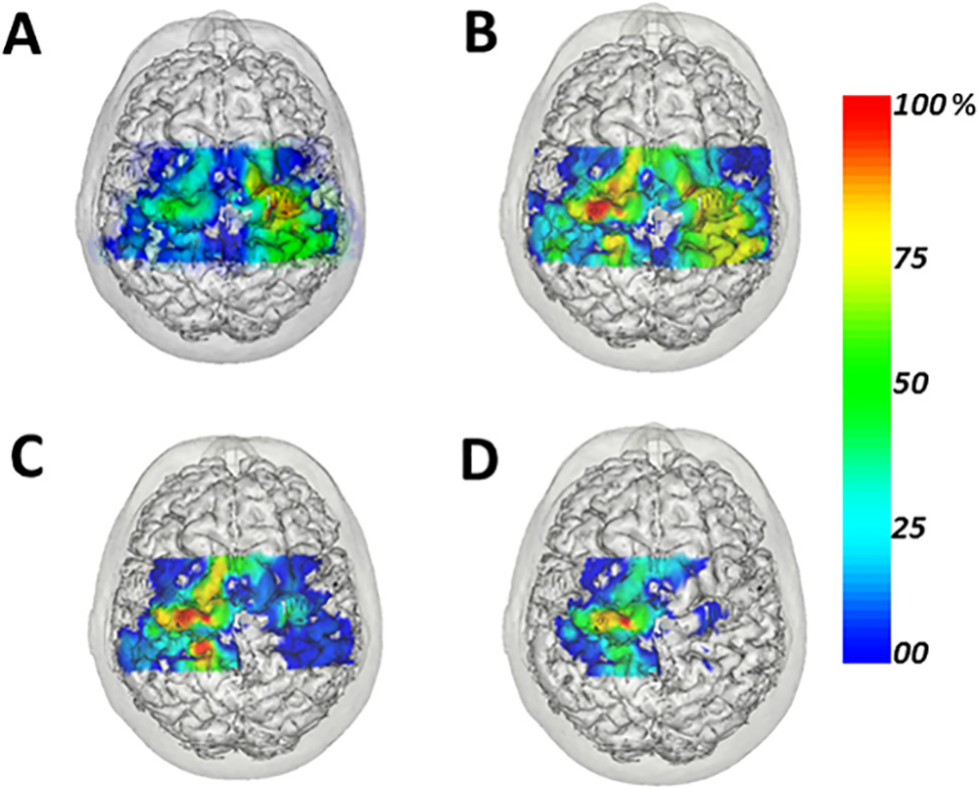
Motor-evoked potential (MEP) amplitudes recorded at each stimulated scalp point and projected on the 3-dimensional brain image of a representative patient (P6)–top view. The amplitude is represented as a percentage of the highest MEP amplitude (100%) at each coil location using a color code from deep-blue (smaller MEP) to red (large MEP). The letters A, B, C and D represent the registered muscles Flexor Digitorum Superficialis (FDS), Abductor Pollicis Brevis (APB), First Dorsal Interosseous (FDI), and Abductor Digiti Minimi (ADM), respectively. For this patient (P6), the left hand was the most affected by leprosy (MAH). Accordingly, in this patient a larger representation map was found for the FDS muscle and smaller representation maps were found for the FDI and ADM muscles in the right in M1, contralateral to the MAH, These results are suggestive of leprosy-induced cortical motor reorganization.

A Wilcoxon test was applied to compare the FDS, APB, FDI and ADM map areas between hemispheres in leprosy patients (N = 4;P3 to P6; S1 Table). Despite the lack of significant differences, smaller motor representation areas were found for the FDI and ADM muscles, innervated by the ulnar nerve, in the hemisphere contralateral to the MAH, as illustrated in Fig 4. In the same vein, no significant differences in overlap of motor representations were found for the four target muscles FDS, APB, FDI and ADM (S2 Table).

### Position of the Center of Gravity (COG)

The position of the center of gravity (COG) of each muscle was compared within and between hemispheres. Differences in the absolute COGs between the hemispheres indicate whether these areas are equidistant from the midline, or whether any asymmetry exists. Moreover, the position of the COGs of each muscle in the same hemisphere might indicate either an overlap or a reorganization of the motor maps. The Wilcoxon test showed no significant differences between hemispheres neither in patients nor in paired controls. Likewise, no significant differences in distance between COGs were observed between the patients and control subjects for all pairs of muscles.

## Discussion

The present study was the first to use transcranial magnetic stimulation (TMS) to examine the organization of the primary motor cortex (M1) of patients diagnosed with leprosy and portraying claw-hand deformity. Results show motor cortical changes in leprosy patients after the so-called "cure" of the Hansen's bacillus infection using multidrug therapy.

The main findings of this study can be summarized as follows. First, grip force assessment showed lower grip strength in the patients’ most affected hand (MAH) compared to the less affected hand (LAH) as well as to the control group. This result is indicative of functional impairment of the patients’ MAH. Moreover, patients had lower resting motor threshold (MT) for the FDS muscle in the hemisphere contralateral to the MAH as compared to the hemisphere contralateral to the LAH. Notwithstanding, higher motor thresholds in both hemispheres were found for this muscle in patients as compared to matched control participants. Finally, motor evoked potential (MEP) amplitudes of the intrinsic hand muscle FDI were lower in the hemisphere contralateral to the MAH as compared to those of the LAH and to the control group. Although the representational motor maps in patients and controls showed great variability, the FDI and ADM muscles in patients tended to have smaller areas in the hemisphere contralateral to the MAH than those obtained in that of the LAH or in matched control subjects. No difference within or between subjects was found for COG and distance between COGs. These results are discussed below.

### Dynamometry (Grip Strength)

Measurement of handgrip strength has gained attention as a simple, non-invasive marker of muscle strength of upper extremities, suitable for clinical use. This assessment reflects the maximum strength derived from combined contraction of extrinsic and intrinsic hand muscles, leading to the flexion of hand joints [55]. Digital or analogical dynamometry has been recommended as an additional method to assess peripheral nerve function in leprosy, particularly in early ulnar impairment [41].

Patients with chronic ulnar/median nerve impairment were herein shown to exhibit a significant decrease in grip strength in the MAH when compared to the LAH as well as to control participants. Accordingly, Rajkumar, Premkumar and Richard [56] observed a high correlation between grip strength and daily life activity in 62 leprosy patients, where patients showing poor results in triple pinch strength could experience more difficulties in daily life activities. Likewise, in the majority of patients in the present study and as attested by the electroneuromyographic evaluation, the ulnar was the most affected nerve, causing weakness in hypothenar muscles and important dysfunctions in the first, fourth and fifth fingers. These effects were clearly most evident for the MAH.

### Corticospinal Excitability and Map Area in Patients with Leprosy

Target muscles (FDS, APB, FDI and ADM) were mapped employing the motor threshold of the FDS muscle at rest (MT). This choice was based on the fact that the median nerve (which supplies the FDS muscle) exhibited control-like ENMG parameters in our cohort (except for P1, see Table 2).

### Higher Motor Threshold (MT) in Leprosy Patients

The FDS muscle MT was globally higher in leprosy patients than in matched controls, with an average difference of 10.4% for the hemisphere contralateral to the MAH and 20.3% for that contralateral to the LAH. Altered MT could result from functional changes at any level of the motor output pathway, thus reflecting changes in the excitability level of neuronal elements within the corticospinal pathway, that is, cortical inhibitory or excitatory interneurons, corticospinal neurons as well as motor units [57,58,59].

Motor neuropathy derived from leprosy is known to impair hand functionality, resulting in grip strength decrease [56] and reduced nerve conduction [45]. These peripheral motor impairments are often accompanied by severe muscle atrophy [60,61,62]. Thus, one could suppose that the higher MT values found in leprosy patients as compared to the control group might reflect enhanced conduction resistance in the motor output pathway.

Besides, changes in the sensory input of leprosy patients with severe sensory loss as abnormalities in the peripheral afferent inputs or in their central processing may interfere with motor output [63]. Indeed, several studies have shown that sensory deprivation resulting from dorsal root or dorsal column transections, skin anesthesia, peripheral neuropathy or inactivation of the somatosensory cortex in humans and non-human primates affect motor behavior [64,65,66,67,68,69,70,71,72] and result in changes in motor cortical representation. Therefore, the complete sensory loss experienced by the leprosy patients, as observed in the ENMG exam, may also be responsible for higher MT. Altogether, these factors may have led to higher MT herein found for the FDS muscle of leprosy patients as compared to control subjects.

### Lower Motor Thresholds and Higher MEP Values in the FDS Muscle: MAH As Compared to the LAH and Control Group

Patients had lower resting motor threshold (MT) for the FDS muscle in the hemisphere contralateral to MAH as compared to the hemisphere contralateral to the LAH. Accordingly, previous studies in humans suffering traumatic amputation of the upper limb showed that the MT of the amputated limb is lower (average 10–17%) in the hemisphere contralateral to the lost limb than that of the ipsilateral hemisphere [29,30,73,74,75,76]. The same holds for individuals who remained immobilized after a fracture of the upper limb [36].

MEP values collected in FDS hotspot in the hemisphere contralateral to the MAH were also higher than those of control subjects, consistent with the observations of Zanette et al, [36]. Higher MEP values and lower MT found in the FDS muscle in the MAH as compared to the LAH indicate that altered handgrip function induced by leprosy can result in a pronounced and long-term reorganization in M1. These results are reinforced by the fact that the median nerve, which exhibited control-like ENMG motor parameters in our cohort, supplies the FDS muscle, from which MT values were collected. Such modifications should thus be rather due to cortical reorganization than to peripheral conduction change induced by leprosy.

### Intrinsic Hand Muscle Reorganization

Smaller MEP amplitudes were found for the FDI muscle of the MAH as compared to the LAH and the control group. Although the map area of the tested intrinsic hand muscles was highly variable both within patients and in control subjects, smaller map areas were also found for the FDI and ADM muscles in the motor cortex contralateral to the MAH as compared to the LAH. These results are in line with those found herein for ENMG and grip strength, and furthers results obtained in hand allograft suggesting that the extent of intrinsic hand muscle representation in M1 associate with hand function [34].

During whole handgrip, the extrinsic muscles provide the major gripping force [77]. The FDS muscle, specifically, seems to be called upon in direct proportion to the required force. Besides, the major intrinsic muscles of whole handgrip are the interosseous, used as phalangeal rotators and metacarpophalangeal flexors [77]. If the function of the interosseous muscles, supplied mostly by the ulnar nerve, is affected by leprosy, then one could suppose that the FDS would take over the handgrip force. Thus, decrease in handgrip force might bear a correlate to the changes in corticospinal excitability shown for FDS (an extrinsic and healthy forearm muscle, being possibly overused due to the chronic dysfunction) and for FDI (an important intrinsic hand muscle affect by ulnar infection). In conclusion, the decrease or loss of sensory afferent neurons and/or an impairment in the strength of peripheral muscles in the ulnar/median territory verified in leprosy patients radically alters the handgrip function leading to cortical motor reorganization in the corresponding affected hand muscles.

### Final Considerations

Leprosy patients usually exhibit a mixed variety of peripheral nerve injuries with sensorimotor impairment, thereby increasing the cortical plasticity challenge and the variability of results (for a discussion, see Reddy et al [20]). Individual factors might however contribute to the consequences of nerve damage. Future studies are needed to fully understand the plastic reorganization in leprosy as well verify cortical motor reorganization after repair procedures.

## Supporting Information

S1 Table Number of active sites at the primary motor cortex for four-target muscles in leprosy patients. Legend: ** not tested; **##** not found; FDS: Flexor Digitorum Superficialis; APB: Abductor Pollicis Brevis; FDI: First Dorsal Interosseous; ADM: Abductor Digiti Minimi; RH: right hemisphere; LH: left hemisphere. In all leprosy patients the left hand was the most affected. The active sites were considered when the motor evoked potential (MEP) value was at least 50μV. Grey color represents subjects enrolled in the statistical analysis.
(DOCX)

S2 Table Overlap of representations considering the four recorded muscles (FDS-APB-FDI-ADM). Legend: ** not tested; **##** not found; "Sites" correspond to the number of overlapping points with simultaneous responses on the four target muscles within each hemisphere. %/Percentage of active sites considering all stimulated points. Grey color represents subjects enrolled in the statistical analysis (FDS: Flexor Digitorum Superficialis; APB: Abductor Pollicis Brevis; FDI: First Dorsal Interosseous; ADM: Abductor Digiti Minimi; RH: right hemisphere; LH: left hemisphere.
(DOCX)
