# Supplementary material for: Primary Motor Cortex Representation of Handgrip Muscles in Patients with Leprosy
Source: PLoS Negl Trop Dis. 2015 Jul 23;9(7):e0003944. doi: 10.1371/journal.pntd.0003944 (PMC4512691; doi:10.1371/journal.pntd.0003944)
Supplement: S1 Table — Legend: ** not tested; ## not found; FDS: Flexor Digitorum Superficialis; APB: Abductor Pollicis Brevis; FDI: First Dorsal Interosseous; ADM: Abductor Digiti Minimi; RH: right hemisphere; LH: left hemisphere. In all leprosy patients the left hand was the most affected. The active sites were considered when the motor evoked potential (MEP) value was at least 50μV. Grey color represents subjects enrolled in the statistical analysis. (DOCX) [file pntd.0003944.s001.docx]

| S1 table. Number of active sites at the primary motor cortex for four-target muscles in leprosy patients. | | | | |  |  |
| --- | --- | --- | --- | --- | --- | --- |
|  | | | | |  |  |
| N=4 | **FDS** | **APB** | **FDI** | **ADM** |  |  |
|  | **RH / LH** | **RH / LH** | **RH / LH** | **RH / LH** |  |  |
| P1 | ** / 24 | ** / ## | ** / ## | ** / ## |  |  |
| P2 | ** / 30 | ** / 30 | ** / 16 | ** / 17 |  |  |
| P3 | 16 / 17 | 23 / 21 | 18 / 21 | 16 / 20 |  |  |
| P4 | 22 /24 | 24 / 39 | 29 / 37 | 24 / 32 |  |  |
| P5 | 21 / 28 | 29 / 32 | 24 / 33 | 19 / 31 |  |  |
| P6 | 21 / 17 | 26 / 27 | 14 / 25 | 01 / 18 |  |  |

Legend: ****** not tested; **##** not found;

FDS: Flexor Digitorum Superficialis; APB: Abductor Pollicis Brevis; FDI: First Dorsal Interosseous; ADM: Abductor Digiti Minimi; RH: right hemisphere; LH: left hemisphere. In all leprosy patients the left hand was the most affected. The active sites were considered when the motor evoked potential (MEP) value was at least 50µV. Grey color represents subjects enrolled in the statistical analysis.
