# Supplementary material for: Primary Motor Cortex Representation of Handgrip Muscles in Patients with Leprosy
Source: PLoS Negl Trop Dis. 2015 Jul 23;9(7):e0003944. doi: 10.1371/journal.pntd.0003944 (PMC4512691; doi:10.1371/journal.pntd.0003944)
Supplement: S2 Table — Legend: ** not tested; ## not found; "Sites" correspond to the number of overlapping points with simultaneous responses on the four target muscles within each hemisphere. %/Percentage of active sites considering all stimulated points. Grey color represents subjects enrolled in the statistical analysis (FDS: Flexor Digitorum Superficialis; APB: Abductor Pollicis Brevis; FDI: First Dorsal Interosseous; ADM: Abductor Digiti Minimi; RH: right hemisphere; LH: left hemisphere. (DOCX) [file pntd.0003944.s002.docx]

|  | S2 table. Overlap of representations considering the four recorded muscles (FDS-APB-FDI-ADM) | |
| --- | --- | --- |
|  |  | |
| N(6) | **RH** | **LH** |
|  | **Sites / %** | **Sites / %** |
| P1 | ** | ## |
| P2 | ** | 14 / 46,6 |
| P3 | 16 / 69,6 | 17 / 80,9 |
| P4 | 20 / 69,0 | 24 / 61,5 |
| P5 | 17 / 58,6 | 25 / 75,7 |
| P6 | 01 / 3,8 | 15 / 55,5 |

Legend: ****** not tested; **##** not found;

"Sites" correspond to the number of overlapping points with simultaneous responses on the four target muscles within each hemisphere. %/Percentage of active sites considering all stimulated points. Grey color represents subjects enrolled in the statistical analysis (FDS: Flexor Digitorum Superficialis; APB: Abductor Pollicis Brevis; FDI: First Dorsal Interosseous; ADM: Abductor Digiti Minimi; RH: right hemisphere; LH: left hemisphere.
